# Orthodontic retreatment of a Class III patient with significant midline asymmetry and bilateral posterior crossbite

**DOI:** 10.1590/2176-9451.20.1.118-126.bbo

**Published:** 2015

**Authors:** Ademir R. Brunetto

**Affiliations:** 1 Specialist in Orthodontics and Facial Orthopedics, University of California. Certified by the Brazilian Board of Orthodontics and Facial Orthopedics (BBO). Former director of the Brazilian Board of Orthodontics and Facial Orthopedics (BBO)

**Keywords:** Angle Class III malocclusion, Dental asymmetry, Posterior crossbite

## Abstract

Posterior crossbite might cause serious long-term functional problems if not early treated. Nevertheless, in older patients, treatment might include palatal expansion in order to correct such malocclusion. In view of the above, this article aims at reporting late correction of bilateral posterior crossbite associated with Angle Class III malocclusion, right subdivision, with consequent midline shift (good skeletal pattern). The case was presented to the Brazilian Board of Orthodontics and Dentofacial Orthopedics (BBO), with DI equal to or greater than 10, as a requirement for the title of certified by the BBO.

## Abstract (pt)

A mordida cruzada posterior pode causar sérios problemas funcionais em longo prazo, se não tratada precocemente. Porém, em alguns pacientes com idade mais avançada, ainda é possível realizar a disjunção palatina para corrigir essa condição. Assim sendo, o objetivo do presente caso é relatar a correção tardia de uma mordida cruzada posterior bilateral, associada a uma má oclusão de Classe III de Angle, subdivisão direita, com consequente desvios de linhas médias dentárias (bom padrão esquelético). Esse caso foi apresentado à Diretoria do Board Brasileiro de Ortodontia e Ortopedia Facial (BBO), se enquadrando na categoria *IGC igual ou superior a 10*, como requisito para revalidação do título de Diplomado.

## INTRODUCTION

**Figure 1 -:**
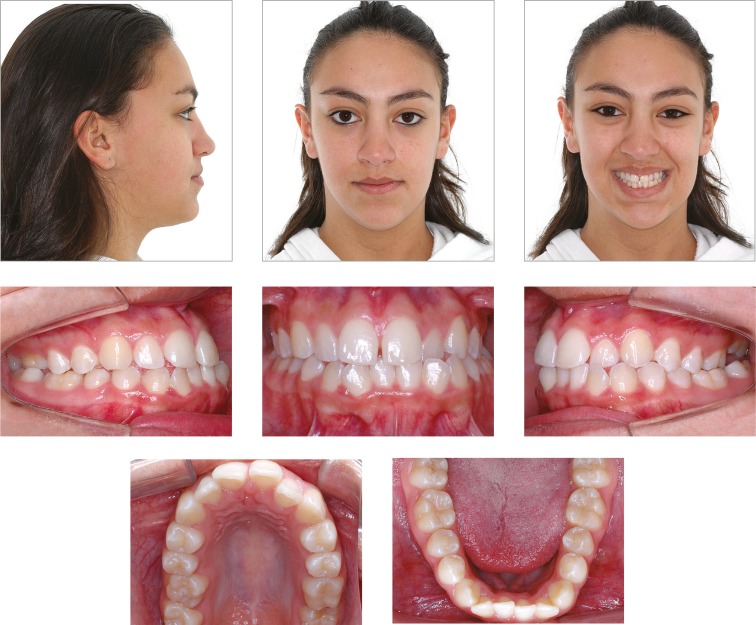
Initial facial and intraoral photographs.

Facial analysis revealed patient's concave profile with everted, slightly forward lower lip in comparison to upper lip, and increased nasolabial angle. In frontal view, she presented mild asymmetry with chin shift to the left associated with slightly increased lower third and passive labial seal. Her smile was significantly and esthetically compromised mainly due to diastema between incisors, lack of coincidence between dental and facial midlines, and large buccal corridor (Fig 1).

Dental analysis revealed a narrow maxillary arch with lack of 1.5 mm for proper alignment, with consequent palatoversion of teeth #16 and 22. Left canine and first molar were more mesially positioned in relation to their counterparts. As for the mandibular arch, posterior teeth presented with lingual inclination of crowns, lack of 3.5-mm space and asymmetry (right canine and first molar more mesially placed). In occlusion, Angle Class III malocclusion, right subdivision, was found in association with bilateral posterior and #22 crossbite. The patient also presented right, 2-mm upper midline shift and left, 2-mm lower midline shift, which resulted in an anesthetic smile. A 2-mm overbite and 1-mm overjet were also found on teeth #21 and 42 (Figs 1 and2).

**Figure 2 -:**
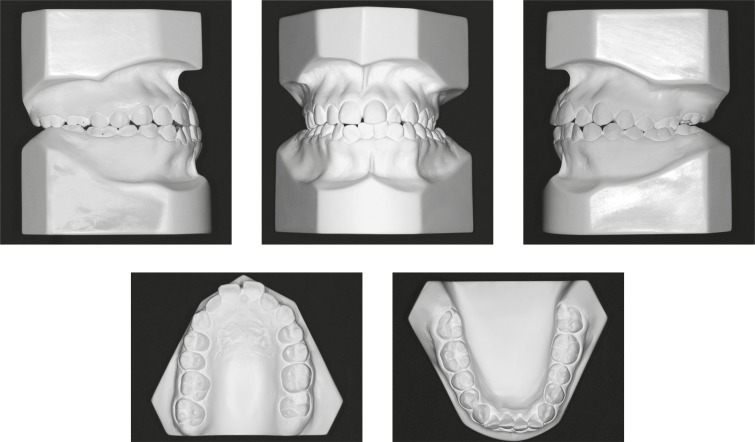
Initial casts.

**Figure 3 -:**
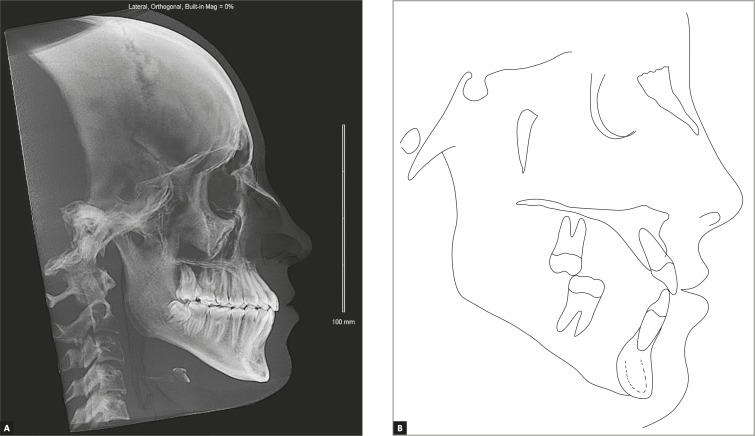
Initial lateral cephalogram (A) and cephalometric tracing (B).

**Figure 4 -:**
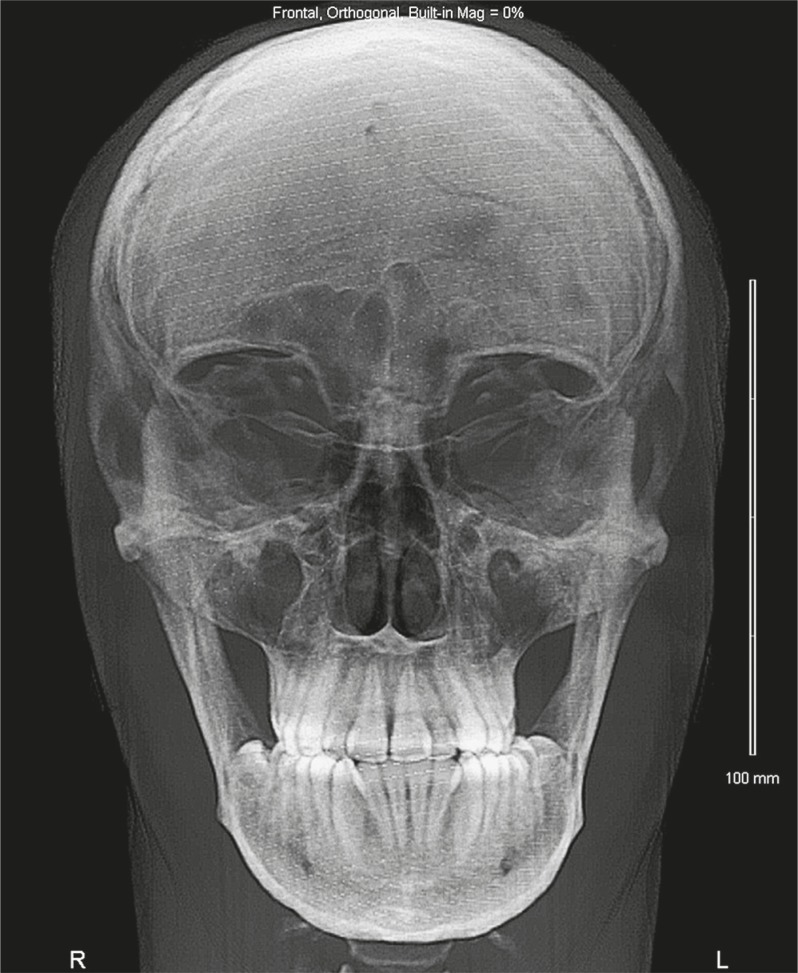
Initial frontal cephalometric radiograph revealing mild iatrogenesis.

**Figure 5 -:**
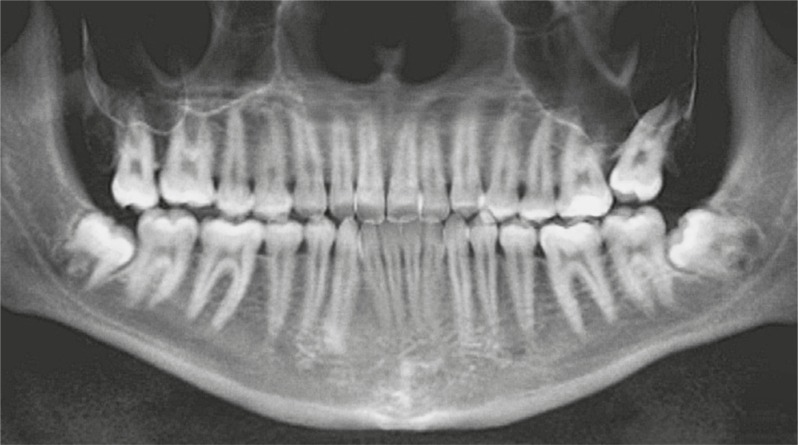
Initial panoramic radiograph

Lateral cephalogram and cephalometric tracing revealed Class I skeletal pattern (ANB = 1^o)^, mild maxillary retrognathism (SNA = 80^o)^ and vertical growth pattern (SN. GoGn = 37^o ^and Y-axis = 56^o)^. From a dental point of view, upper incisors were slightly proclined and protruded, while lower incisors were properly positioned (Fig 3 andTable 1). Facial asymmetry was evident in frontal radiograph which revealed mild mandibular deviation to the left due to asymmetric condyle growth (Fig 4). Panoramic radiograph raised the initial possibility of ankylosis of #16, which was further denied by infraocclusion. Tooth #27 was absent and had been replaced, in position, by #28 which, in turn, was also in infraocclusion. The patient was in good periodontal health and had #18 missing (Fig 5).

**Table 1 -:** Initial (A) and final (B) cephalometric values.

	Measurements		Normal	A	B	Dif. A/B
Skeletal pattern	SNA	(Steiner)	82°	80°	80°	0
	SNB	(Steiner)	80°	81°	78.5°	2.5
	ANB	(Steiner)	2°	-1°	1.5°	2.5
	Angle of convexity	(Downs)	0°	-3.5°	1.5°	5
	Y axis	(Downs)	59°	56°	61°	5
	Facial angle	(Downs)	87°	90°	88°	2
	SN-GoGn	(Steiner)	32°	37°	39°	2
	FMA	(Tweed)	25°	27°	28°	1
Dental pattern	IMPA	(Tweed)	90°	86°	96°	10
	1.NA (degrees)	(Steiner)	22°	32°	33°	1
	1-NA (mm)	(Steiner)	4 mm	7 mm	8 mm	1
	1.NB (degrees)	(Steiner)	25°	23°	31°	8
	1-NB (mm)	(Steiner)	4 mm	5 mm	6 mm	1
	- Interincisal angle	(Downs)	130°	130°	120°	10
	1-APo	(Ricketts)	1 mm	4 mm	5.5 mm	1.5
Profile	Upper lip — S-line	(Steiner)	0 mm	-1.5 mm	0 mm	1.5
	Lower lip — S-line	(Steiner)	0 mm	2 mm	1 mm	1

**Table 2 -:** Model measurements.

Measurements	A	B	Dif. A/B
Distance between upper canines	37	38	1
Distance between lower canines	27	29	2
Distance between upper molars	46	51	5
Distance between lower molars	44	46	2

## TREATMENT PLAN

Taking patient's most significant problems into account, treatment plan included palatal expansion with Haas appliance initially activated 2/4 of a turn a day followed by 1/4 a day until overcorrection was achieved. After a 120-day retention period, fixed appliance was mounted on the upper and mandibular arches with 0.022 x 0.028-in preadjusted, Edgewise slots. Initial alignment and leveling were performed by means of the following sequence of NiTi archwires: 0.012-in, 0.016-in, 0.016 x 0.022-in and 0.017 x 0.025-in on both upper and mandibular arches. Subsequently, mechanics with 1/4-in intermaxillary Class III elastics (200 g/force) associated with jig and NiTi springs was applied on one side. Once dental and facial midlines were coinciding, the finishing phase was started with ideal 0.019 x 0.025-in stainless archwires, with lingual torque applied to upper anterior teeth and buccal torque applied to second molars. The patient was then referred to lower third molars extraction.

Alternative treatment plan included the use of mini-implants in the upper left and lower right hemi-arches for canine distalization so as to achieve arch symmetry and midline coincidence. Another option would include surgical maxillary advancement for sagittal discrepancy correction. Taking the cost-benefit relationship of treatment planning into account, we initially opted for conservative treatment.

## TREATMENT PROGRESS

Palatal expansion achieved satisfactory results, with mid palatal suture opening without excess compensatory proclination of upper posterior teeth. Once the expansion appliance was rendered stable and a 120-day retention period had passed, the sequence of archwires established at initial treatment planning was followed. Tooth #16, initially under suspicion of ankylosis, responded well to orthodontic forces. Patient's compliance was satisfactory with regard to the use of elastics, thereby increasing the possibilities of achieving acceptable occlusion. 

## RESULTS

**Figure 6 -:**
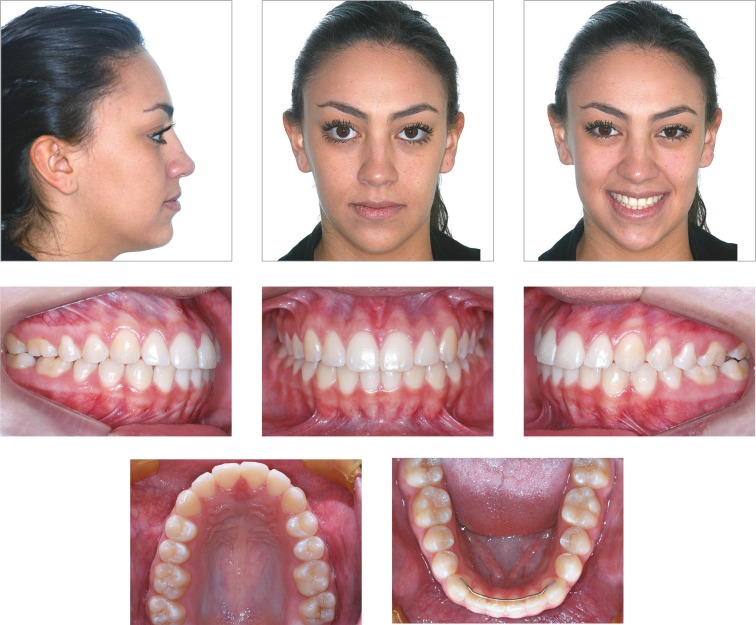
Final facial and intraoral photographs.

Upper lip protrusion led to a balanced facial profile as it was positioned slightly forward with decreased nasolabial angle. In frontal view, protrusion resulted in significant facial improvements with increased nasolabial fold. At smiling, the patient evinced a decreased buccal corridor which, together with midline coincidence, also led to significant esthetic changes (Fig 6). 

Upper dental arch analysis revealed satisfactory alignment achieved as a result of increased arch circumference that, in turn, led to expansion. Teeth #16 and 28, previously under infraocclusion, were duly aligned. Right canine underwent mesialization which improved intra-arch anteroposterior symmetry. In the mandibular arch, satisfactory alignment and leveling were achieved by means of torque correction in lower posterior teeth as a result of palatal expansion. Right canine distalization achieved by means of Class III elastics on one side also aided to create space necessary to correct model discrepancy. In terms of function, molar and canine relationship was achieved on both sides, with ideal lateral and anterior disocclusion. In addition to midline coincidence, overjet and overbite were within normal standards (Figs 6 and7).

**Figure 7 -:**
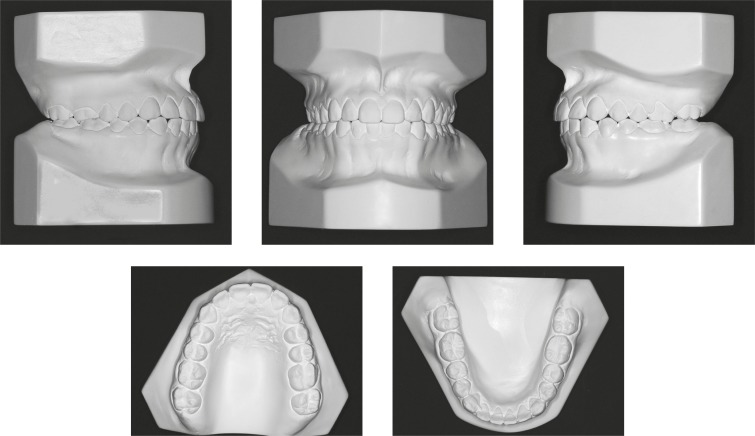
Final casts.

**Figure 8 -:**
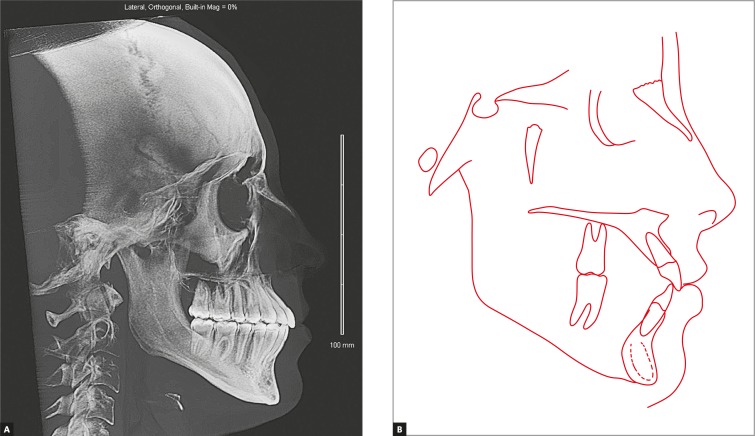
Final lateral cephalogram (A) and cephalometric tracing (B). A B

**Figure 9 -:**
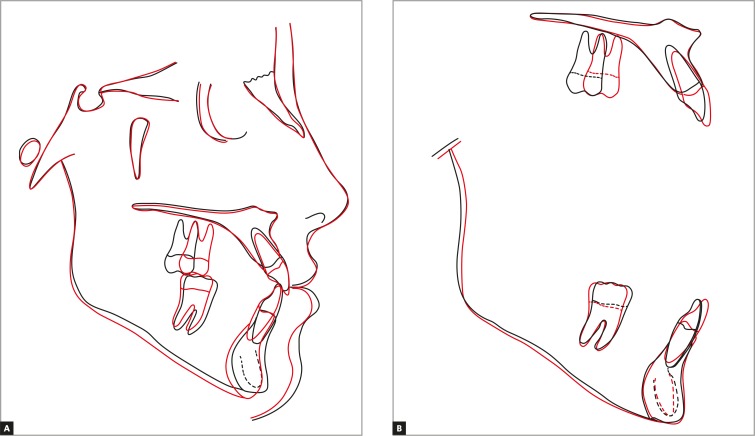
Total (A) and partial (B) initial (black) and final (red) cephalometric tracings superimposition

Cephalometry revealed decreased SNB and, as a consequence, ANB angle due to slight clockwise rotation of the mandible evinced by an increase of 2^o ^in the Sn.GoGn angle. Upper incisors were slightly proclined, unlike lower incisors in which proclination was much more significant. There was upper lip protrusion and lower lip setback, both of which were responsible to establish facial profile balance (Fig 8,Tab 1). In addition, there was significant change in the distance between upper molars (an increase of 5 mm) proving greater palatal opening in the posterior region. Total cephalometric superimposition evinced clockwise rotation of the mandible and posterior displacement of the lower lip. Partial superimposition of maxillary segments revealed significant posterior anchorage loss (on the right side) and slight extrusion and proclination of incisors. Conversely, partial superimposition of mandibular segments revealed slight distalization of molars (on the right side) and proclination of incisors (Fig 9). 

**Figure 10 -:**
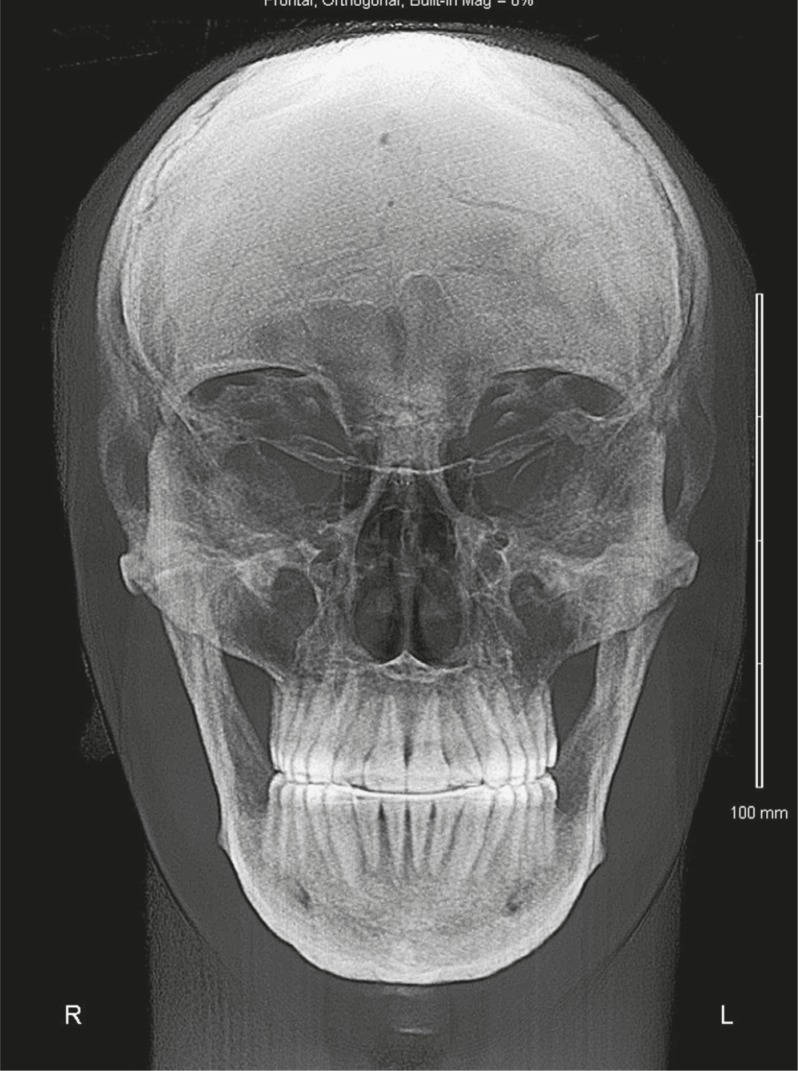
Final frontal cephalometric radiograph

**Figure 11 -:**
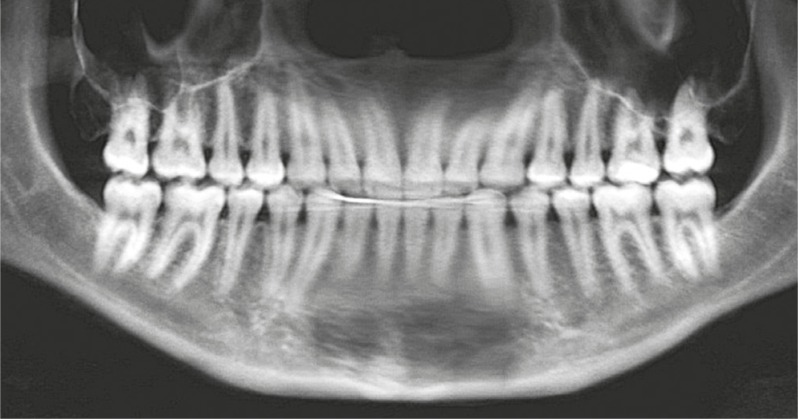
Final panoramic radiograph.

Frontal cephalogram showed that mandibular shift to the left remained stable after maxillary expansion, while midlines were corrected so as to coincide with the median sagittal plane (Fig 10). Panoramic radiograph not only revealed good periodontal health after orthodontic movement, but also satisfactory root parallelism in both the maxilla and mandible. Tooth #28 was positioned slightly upward and distally, which is considered ideal; however, it should undergo natural movement, including more physiological movements, so as to be better positioned in line of occlusion (Fig 11).

## FINAL CONSIDERATIONS

Maxillary expansion is ideally performed when the mid palatal suture is not yet mature and does not present interdigitation, which occurs before patients achieve pubertal growth spurt.^1^^-^ 4 The younger the patient is, the greater the orthopedic component and the smaller the chances of relapse. Nevertheless, in some cases, patients have already achieved this stage of growth, but might as well satisfactorily respond to forces applied to the palatal suture despite greater bone density and interdigitation. Capelloza Filho et al^5^found a success rate of 81.5% in expansion of adult patients. On the other hand, the technique might produce more severe deleterious effects in adults than in children and adolescents, including edema, buccal clinical attachment loss and occlusal plane instability.^5^^,^ 6 Thus, the procedure needs to be carefully performed and strictly followed by a professional. Nevertheless, it should always be considered, since it potentially prevents surgery (LeFort I osteotomy).^7^

There is also some discussion on the amount of anterior and posterior opening and in which proportion they occur. In the case reported herein, we noticed greater opening in the posterior region, which was evinced by an increase of 5 mm in the distance between molars despite significant anchorage loss of #16. This finding is in accordance with most studies.^8 ^The literature also reports clockwise rotation of the mandible as a consequence of palatal expansion, which is caused due to extrusion of palatal cusps in anchorage teeth.^9^^,^ 10 ^,^ 11 In the case reported herein, we found clockwise rotation of the mandible leading to slight increase in lower facial height; however, without causing significant esthetic damage to the face and, therefore, being clinically irrelevant.^12 ^Additionally, such mandibular movement is likely to recede in the long-term.^13^

We opted for mechanics with Class III elastics and jig/springs on one side due to opposite shift of upper and lower midlines. When applied to one side only, this mechanics not only promotes mesialization of upper posterior teeth, but also distalization of lower posterior teeth (highly evident in partial cephalometric superimposition).^14^^,^ 15 Movement of the maxilla and mandible in opposite directions favors simultaneous correction of dental midlines, causing them to coincide with the facial midline. However, long-term use of intermaxillary elastics on one side should be avoided, as it may lead to imbalance of stomatognathic system muscles and, as a result, temporomandibular disorders, migraine or local pain. Control should be ongoing, and should any of the aforementioned factors be identified, mechanics should be immediately removed. Despite being a common fact, symptoms should never be underestimated by the orthodontist, as it might hinder treatment assessment.^16^

In addition to patient's advanced age, previous use of fixed orthodontic appliance was also considered a complicating factor. Whenever teeth, periodontal ligament and alveolar bone have already been subject to non-physiological forces, additional care should be taken, particularly with regard to the magnitude of orthodontic forces applied. Consensus has been reached on the fact that treatment duration and magnitude of forces play an important role in triggering orthodontically induced root resorption.^17^^-^ 20 In the present study, total treatment time (36 months) increased due to patient's absence, despite cooperation on the use of intermaxillary elastics.

Lastly, therapy fulfilled treatment objectives without subjecting the patient to invasive procedures, such as orthognathic surgery. There was significant improvement in facial profile at rest and at smile, and all requirements necessary for functional and stable occlusion were met.
